# Immunomodulatory activity of *Lactobacillus plantarum* KLDS1.0318 in cyclophosphamide-treated mice

**DOI:** 10.29219/fnr.v62.1296

**Published:** 2018-03-21

**Authors:** Yueyue Meng, Bailiang Li, Da Jin, Meng Zhan, Jingjing Lu, Guicheng Huo

**Affiliations:** Key Laboratory of Dairy Science, Ministry of Education, Northeast Agricultural University, Harbin, China

**Keywords:** Lactobacillus plantarum KLDS1, 0318, splenocyte proliferation, NK cell activity, macrophages phagocytosis, cytokine

## Abstract

**Background:**

Probiotics in fermented foods have attracted considerable attention lately as treatment options for immune diseases, the incidence of which has been increasing throughout the world.

**Objective:**

The objective of the present study was to investigate the immunomodulatory activity of *Lactobacillus plantarum* (*L. plantarum*) KLDS1.0318 in cyclophosphamide-treated mice.

**Design:**

To investigate the immune-enhancing effects of *L. plantarum* KLDS1.0318, we used a immunosuppressive model. Ninety female six-week-old BALB/c mice were randomly divided into six groups: normal control (NC) group, model control (MC) group, immunosuppression plus *L. plantarum* KLDS1.0318 groups with three different doses (KLDS1.0318-L, KLDS1.0318-M, and KLDS1.0318-H), and plus levamisole hydrochloride as positive control (PC) group.

**Results and discussions:**

Results showed that the thymus and spleen indexes of the four treatment groups were significantly higher than those of the MC group (2.01±0.16) ( *p* < 0.05). The capacity of lymphocyte proliferation, the activity of natural killer (NK) cell and macrophages phagocytosis were significantly increased ( *p* < 0.05) in four treatment groups as compared with the MC group (0.327±0.022, 62.29±0.8, 0.087±0.008, respectively). The levels of relative immune factors (IL-2, IL-6, and IFN-γ) showed similar patterns ( *p* < 0.05).

**Conclusions:**

This study suggested that orally administered L.plantarum KLDS1.0318 may effectively accelerate the recovery of immunosuppressive mice caused by cyclophosphamide (CTX). The immunomodulatory activity of the srtain recommended that L. plantarum KLDS1.0318 could be used as a powerful medicinal treatment against immunosuppression.

Immunosuppression is a state of temporary or long-lasting immunity dysfunction that results in making the organism more sensitive to pathogens because of the impairment of the immune system (1–4). For example, the human immunodeficiency virus epidemic caused one of the most significant populations of immunocompromised hosts (5), which often brings about a low antibody level or ineffective vaccination in a vaccinated host. For the purpose of controlling viral infectious diseases and preventing secondary infection, vaccines and immunopotentiating drugs, such as levamisole at a high dose, have to be used for long-term cure, which often leads to a great deal of side effects, such as serious neurological symptoms, gastric hemorrhage, colic, anemia, and vasculitis (6–8). Accordingly, investigating and developing new and safer immunomodulating agents is one of the most effective and efficient methods for prevention and treatment of immunosuppressive diseases (9).

People have been seeking an effective means to prevent and remedy immunosuppressive diseases for years, but the progress is slow. In the meantime, the application of traditional probiotics in immunoregulation has acquired some achievements. Probiotics have complicated nutritional requirements and are found in a variety of habitats, such as human and animal mucosal membranes, material of plant origin, sewage, and fermented dairy products and spoiled food (10). They play a vital role in immunomodulation, maintaining the intestinal microbial balance, and preventing gastrointestinal infection. Previous research indicated that lactobacilli can be used for immune stimulation to increase early lines of defense against invading harmful bacteria (11).

Lactobacilli are able to promote immunity in mice, and this effect is dose and strain reliant (12, 13). As the expression profiles of cell wall proteins and content of DNA unmethylated cytidine guanine dinucleotide varied in different probiotics, different probiotic strains and dosages may bring about different immune responses (14–18). A great many studies have reported that *Lactobacillus plantarum* has immunoregulatory function: activation of Th1 immune responses (19), promotion of IgA secretion and prevention of influenza virus infection (20), enhancement of the cytokine profile against mite allergy (21), and improvement of natural killer (NK) cell activity, for instance (22).

*L. plantarum* KLDS1.0318, a newly identified probiotic, was preserved in our laboratory. Its effects on the activity of immune cells *in vitro* were previously investigated and it is considered to be possessed of a higher immunomodulatory activity (23). However, the immunoregulatory effects of *L. plantarum* KLDS1.0318 *in vivo* are not fully clear yet and neither is its immunoregulatory mechanism.

Cyclophosphamide (CTX), a classical myelotoxic agent, was used in a previous study to establish an experimental model applicable to the evaluation of immunomodulation by antibiotics in normal and immunocompromised mice (24). The aim of this experiment was to establish an immunosuppressive model by treating BALB/c mice with CTX. Using this model, the possible effects of *L. plantarum* KLDS1.0318 on the immune system of immunocompromised hosts were investigated.

## Materials and methods

### Experimental animals

Ninety female-specific pathogen-free BALB/c mice with a body weight of 20.0 ± 2.0 g were obtained from Beijing Vital River Laboratory Animal Technology Co., Ltd. (Beijing, China). Animals were acclimatized to laboratory conditions for 1 week before commencement of the animal experiment. They were housed in plastic cages at an ambient temperature of 23 ± 1°C, 50 ± 10% humidity, and a 12/12 h light–dark cycle, fed standard laboratory chow, and allowed water *ad libitum*. Animals used in this study were cared for in accordance with the *Guide for the Care and Use of Laboratory Animals* published by the US National Institutes of Health (NIH Publication 85-23, 1996), and all experimental procedures were approved by the Animal Care Review Committee, Northeast Agricultural University.

### Preparation of bacterial strain

*L. plantarum* KLDS1.0318 (preserved at Key Laboratory of Dairy Science, Ministry of Education, Northeast Agricultural University) was grown in de Man, Rogosa & Sharpe (MRS) medium (peptone 10.0 g, beef extract 10.0 g, glucose 20.0 g, yeast extract powder 5.0 g, sodium acetate 5.0 g, dipotassium hydrogen phosphate 2.0 g, triammonium citrate 2.0 g, magnesium sulfate 0.5 g, manganese sulfate 0.05 g, Tween 80 1.0 g, distilled water 1,000 mL, pH 6.5) (25) for 18 h at 37 °C. Bacteria were subcultured twice before inoculation of the batch culture at 10^7^ colony-forming units (CFUs)/mL. For the preparation of gavages, the bacteria were harvested by centrifugation (2,000 × g, 10 min), washed twice in sterile phosphate-buffered saline (PBS), and resuspended in sterile PBS. In the pre-experiment, for the assessment of approximate concentrations of viable bacteria, suitable dilutions of the culture were plated onto MRS broth at 37°C for 48 h. The concentrations of *L. plantarum* KLDS1.0318 were found to reach 5 × 10^9^ CFU/mL. The bacterial strain was diluted in sterile PBS to produce suspensions of designated doses for oral administration.

### Chemicals

Roswell Park Memorial Institute (RPMI) 1640, fetal bovine serum (FBS), concanavalin A (ConA), and levamisole hydrochloride were purchased from Sigma Co. (St. Louis, MO, USA). Enzyme-linked immunosorbent assay (ELISA)–based cytokine kits were purchased from Cloud-Clone Corp. (Houston, TX, USA). CTX was purchased from Beijing Solarbio Science & Technology Co., Ltd. (Beijing, China).

### Experimental design

All mice were randomly divided into six groups: a normal control (NC) group, model control (MC) group, three *L. plantarum* KLDS1.0318 groups with different doses (KLDS1.0318-L, 5 × 10^7^ CFU/mL; KLDS1.0318-M, 5 × 10^8^ CFU/mL; KLDS1.0318-H, 5 × 10^9^ CFU/mL, 0.2 mL/d), and levamisole hydrochloride (40 mg/kg) as a positive control (PC) group. Except the NC group, the other five groups were injected intraperitoneally with CTX 80 mg/kg/d of body weight in sterile saline for three consecutive days to induce immunosuppression. Body weight was used as a measure of immunosuppression effect (26). All treatments were conducted with 10 mL/kg body weight by oral administration once daily for 20 days. The NC group mice were injected and received an equivalent volume of sterile PBS as the immunosuppression group. An equivalent volume of sterile PBS was administered to MC group mice in the same way.

## Analysis of body weight

Animal body weight was monitored every 4 days throughout the experiment.

## Analysis of immune organ index

The mice were weighed before being sacrificed 20 days after the commencement of oral administration. The thymus and spleen were immediately excised surgically and weighed. The immune organ index was calculated according to the following formula:

$$\text{spleen or thymus indices}\left(\text{mg}/\text{g}\right)=\frac{spleenorthymusweight(mg)}{bodyweight(g)}$$ (27) .

### Assay of splenocyte proliferation induced by T-cell mitogen conA

Mouse spleens were aseptically removed, placed in 0.1 M cold PBS, gently homogenized, and passed through a 200-mesh sieve to generate single-cell suspensions, as previously described (28). Erythrocytes were rapidly washed by hypoosmotic hemolysis. Next, the cells were suspended at a final density of 1 × 10^6^ cells/mL in RPMI 1640 medium supplemented with 10% FBS. Splenocytes were placed into 96-well flat-bottomed microplates in triplicate at 2 × 10^5^ cells/well, and then 2.5 μg/well of conA was added to the wells. The cells were then cultured at a total volume of 200 μL/well at 37°C in 5% CO_2_. Serum-free RPMI 1640 medium was used as the control. After 48 h of incubation, 20 μL CCK-8 (Dojindo Laboratories, Kumamoto-ken, Japan) was added to each well and the plate was incubated for another 2.5 h. Finally, the absorbance at 450 nm was measured using a microplate reader (XD711, Shanghai Xunda Medical Instrument Co., Ltd., Shanghai, China).

### Assay of NK cell activity

NK cell activity was determined using a CCK-8 assay kit (Dojindo Laboratories). Splenocytes were prepared as section Assay of splenocyte proliferation induced by T-cell mitogen conA. Briefly, blank control (RPMI 1640) and spleen cells (1 × 10^6^ cells/mL) were added at the level of 0.1 mL per well. One hundred microliters of 1 × 10^4^ cells/mL YAC-1 cells, used as the target cells, were added into the wells as mentioned above, RPMI 1640 and spleen cells were added at 0.1 mL per well, used as the effector cells. The plates were then incubated at 37°C in 5% CO_2_ for 20 h. Next, 20 μL of CCK-8 was added. Following another 4 h of co-culture, the optical density of each well was measured using an XD711 microplate reader (Shanghai Xunda Medical Instrument Co., Ltd.). In addition, absorbance measurements were also recorded for the target cell control, blank control, and effector cell control. The percentage of NK cell activity was determined by the following equation:

$$\%\text{ of NK cell activity}=(1-\frac{opticaldensityvalueoftestsamples-opticaldensityvalueofeffectorcellcontrol}{opticaldensityvalueoft\arg etcellcontrol})\times 100$$ (29)

### Determination of pinocytosis of peritoneal macrophages

Mice were sacrificed and peritoneal cells were harvested by peritoneal lavage with 4 mL of RPMI 1640 medium supplemented with 10% heat-inactivated FBS. Three milliliters of cell-rich lavage fluid was aspirated and centrifuged at 1,500 rpm for 5 min. The pellet was resuspended at 1 × 10^6^ cells/mL in RPMI 1640 medium and seeded in 96-well plates at 200 μL/well (30). The plates were then incubated for 3 h at 37°C in 5% CO_2_, washed three times, and nonadherent cells were removed by aspiration. Attached cells were used as peritoneal macrophages (31). The cells were resuspended in 200 μL RPMI 1640 containing 10% FBS. After 24 h culture at 37°C under 5% CO_2_, the culture medium was discarded and 100 μL of 0.072% neutral red was added to each well and cultured for another 0.5 h. Then, the mixed solution was discarded and each well was washed thrice with PBS buffer to remove the excess dye and was blotted dry. The cells were resuspended in 50% ethanol containing 1% glacial acetic acid (lysis solution) and maintained overnight at 4°C. Optical densities were then read at 540 nm and expressed as the phagocytosis index.

### Cytokine quantitation

To measure IL-2, IL-6, and IFN-γ by ELISA, the blood of each mouse was collected from the orbital cavity under diethyl ether anesthesia. The fresh blood was kept standing for 10 min at 37°C and then for 15 min at 4°C. Serum was obtained by centrifugation at 3,000 rpm for 10 min and then stored at −40°C until use (30). Concentrations of cytokines (IL-2, IL-6, and IFN-γ) in serum were determined using ELISA assay kits (Cloud-Clone Corp.), according to the manufacturer’s instructions. The results were expressed as the concentration of cytokines per milliliter of mouse serum by standard cytokines provided in the kits.

## Statistical analysis

Results were expressed as mean ± standard deviation (SD) of at least three replicates, and the data were analyzed with SPSS 20.0 software (SPSS Inc., Chicago, IL, USA). The statistical significance of data comparisons was determined using one-way analysis of variance, followed by Duncan’s multiple range test. Values of *p* < 0.05 were considered to be statistically significant.

## Results

### Body weight

**Fig. 1:**
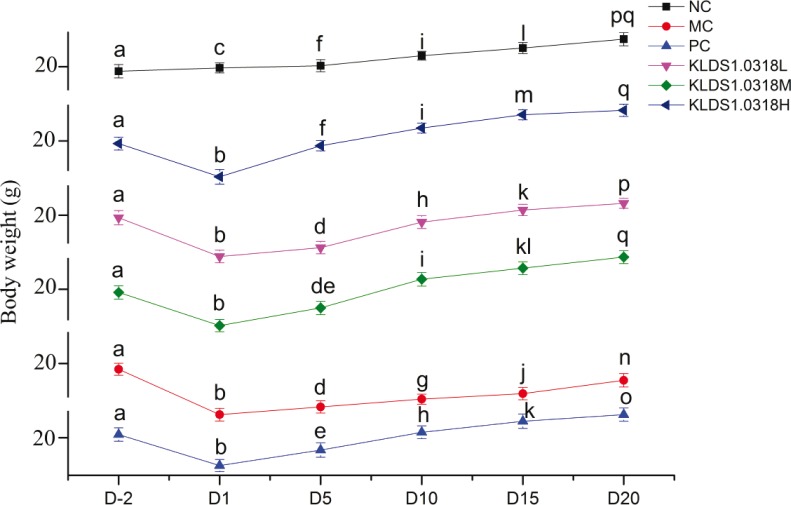
Changes of body weight in the six groups of mice. NC, non-immunosuppression + sterilized PBS; MC, immunosuppression (IM) + sterilized PBS; PC, IM + levamisole hydrochloride (40 mg/kg); KLDS1.0318-L, IM + 5 × 10^7^ CFU/mL *Lactobacillus plantarum* KLDS1.0318; KLDS1.0318-M, IM + 5 × 10^8^ CFU/mL *L. plantarum* KLDS1.0318; KLDS1.0318-H, IM + 5 × 10^9^ CFU/mL *L. plantarum* KLDS1.0318. Data are expressed as the mean ± SD (*n* = 15). Different letters represent significant differences between groups in the same time point (*p* < 0.05). NC, normal control; PBS, phosphate-buffered saline; MC, model control; PC, positive control; CFU, colony-forming units.

Animal body weight of all the groups was monitored six times in total throughout the experiment. As shown in Fig. 1, there were no big differences in initial body weights on Day 2, after acclimatization in the animal house for 1 week. Subsequently, all five immunosuppressed mice groups, compared with the NC group, showed a dramatic decrease in body weight following the injection of CTX (*p* < 0.05). Compared with the MC group, the KLDS1.0318-L, KLDS1.0318-M, KLDS1.0318-H, and PC groups exhibited more body weight gain throughout the remaining experimental period.

## Immune organ index

As shown in Table 1, compared with the MC mice, the thymus indexes in the PC, KLDS1.0318-M, and KLDS1.0318-H groups were significantly improved (*p <* 0.05); however, there was no significant difference in the low dose of KLDS1.0318 (*p* > 0.05). As for spleen indexes, they were significantly increased (*p <* 0.05) in each KLDS1.0318 treatment group and PC group as compared with those of the MC group. In addition, KLDS1.0318 treatment exhibited a stronger effect on the thymus index than that of the levamisole hydrochloride–treated mice at a dose of 5 × 10^9^ CFU/mL (0.2 mL/d) (*p <* 0.05).

**Table 1:** Effect of *Lactobacillus plantarum* KLDS1.0318 on thymus and spleen indices in mice

Group	Thymus index (mg/g)	Spleen index (mg/g)
NC	2.67 ± 0.22^b^	5.83 ± 0.19^d^
MC	2.01 ± 0.16^a^	4.44 ± 0.17^a^
PC	2.75 ± 0.15^b^	5.56 ± 0.21^cd^
KLDS1.0318-L	2.20 ± 0.11^a^	4.81 ± 0.16^b^
KLDS1.0318-M	2.60 ± 0.20^b^	5.24 ± 0.19^c^
KLDS1.0318-H	3.15 ± 0.17^c^	5.63 ± 0.24^d^

NC, non-immunosuppression + sterilized PBS; MC, immunosuppression + sterilized PBS; PC, IM + levamisole hydrochloride (40 mg/kg); KLDS1.0318-L, IM + 5 × 10^7^ CFU/mL *L. plantarum* KLDS1.0318; KLDS1.0318-M, IM + 5 × 10^8^ CFU/mL *L. plantarum* KLDS1.0318; KLDS1.0318-H, IM + 5 × 10^9^ CFU/mL *L. plantarum* KLDS1.0318. Data are expressed as the mean ± SD (*n* = 15). Significant differences (*p* < 0.05) between the groups are indicated with different letters above the data.

### Effect of L. plantarum KLDS1.0318 on conA–induced lymphocyte proliferation

**Fig. 2:**
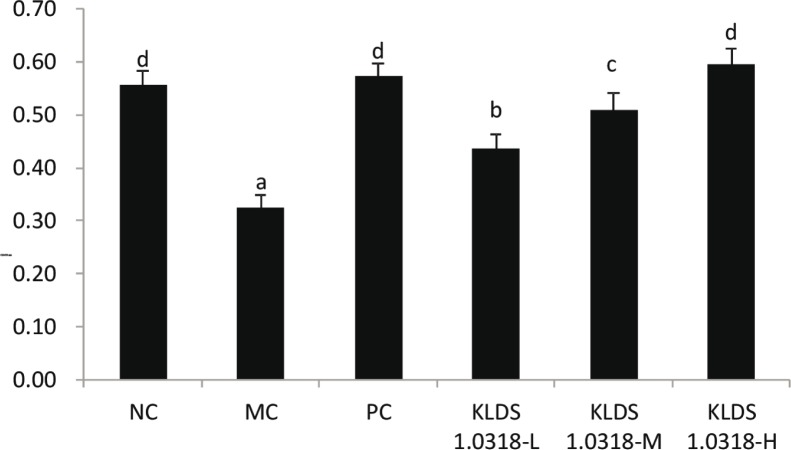
Effect of *L. plantarum* KLDS1.0318 on lymphocyte proliferation in mice. NC, non-immunosuppression + sterilized PBS; MC, immunosuppression + sterilized PBS; PC, IM + levamisole hydrochloride (40 mg/kg); KLDS1.0318-L, IM + 5 × 10^7^. CFU/mL *L. plantarum* KLDS1.0318; KLDS1.0318-M, IM + 5 × 10^8^ CFU/mL *L. plantarum* KLDS1.0318; KLDS1.0318-H, IM + 5 × 10^9^ CFU/mL *L. plantarum* KLDS1.0318. Data are expressed as the mean ± SD (*n* = 15). Significant differences (*p* < 0.05) between the groups are indicated with different letters above the bars.

The effect of *L. plantarum* KLDS1.0318 on the proliferation of splenic T lymphocytes is shown in Fig. 2. The spleen lymphocyte proliferation capacity was significantly increased in the KLDS1.0318-treated groups when compared with the MC group (*p* < 0.05). Moreover, each KLDS1.0318-treated group showed a large increase in a dose-dependent manner, and the change was significant (*p* < 0.05). There was no significant difference in the NC, PC, and KLDS1.0318-H groups (*p* > 0.05). The results indicated that *L. plantarum* KLDS1.0318 could stimulate a T-lymphocyte–specific proliferative response.

### Effect of L. plantarum KLDS1.0318 on NK cell activity

**Fig. 3:**
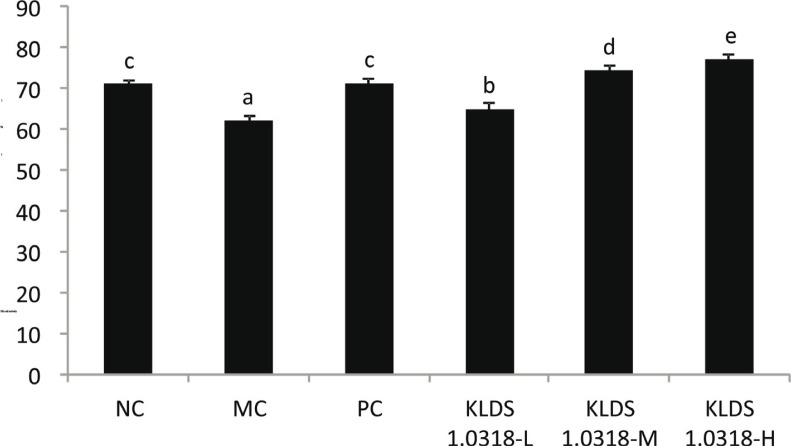
Effect of *L. plantarum* KLDS1.0318 on NK cell activity in mice. NC, non-immunosuppression + sterilized PBS; MC, immunosuppression + sterilized PBS; PC, IM + levamisole hydrochloride (40 mg/kg); KLDS1.0318-L, IM + 5×10^7^ CFU/mL *L. plantarum* KLDS1.0318; KLDS1.0318-M, IM + 5×10^8^ CFU/mL *L. plantarum* KLDS1.0318; KLDS1.0318-H, IM + 5×10^9^ CFU/mL *L. plantarum* KLDS1.0318. Data are expressed as the mean ± SD (*n* = 15). Significant differences (*p* < 0.05) between the groups are indicated with different letters above the bars.

As shown in Fig. 3, the results showed that KLDS1.0318 treatment significantly improved (*p* < 0.05) NK cell activity in mice (L, M, H) in a dose-dependent manner. Levamisole hydrochloride treatment also exhibited strong effects on NK cell activity in the PC group mice in comparison with the MC group (*p* < 0.05). Moreover, there was no significant difference between the NC and PC groups (*p* > 0.05). In addition, it was found that *L. plantarum* KLDS1.0318 treatment exhibited a stronger effect on NK cell activity than the PC (levamisole hydrochloride–treated mice) group at a dose of 5 × 10^8^ and 5 × 10^9^ CFU/mL (0.2 mL/d) (*p <* 0.05).

### Effect of L. plantarum KLD1.0318 on phagocytic activity of macrophages

**Fig. 4:**
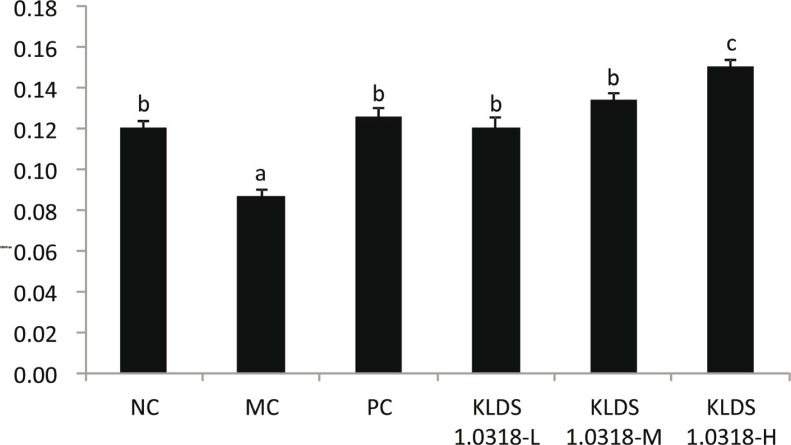
Effect of *L. plantarum* KLDS1.0318 on phagocytic activity of macrophages in mice. NC, non-immunosuppression + sterilized PBS; MC, immunosuppression + sterilized PBS; PC, IM + levamisole hydrochloride (40 mg/kg); KLDS1.0318-L, IM + 5 × 10^7^ CFU/mL *L. plantarum* KLDS1.0318; KLDS1.0318-M, IM + 5 × 10^8^ CFU/mL *L. plantarum* KLDS1.0318; KLDS1.0318-H, IM + 5 × 10^9^ CFU/mL *L. plantarum* KLDS1.0318. Data are expressed as the mean ± SD (*n* = 15). Significant differences (*p* < 0.05) between the groups are indicated with different letters above the bars.

On Day 20, after oral administration of KLDS1.0318 to CTX-treated mice, we isolated the macrophages and examined their phagocytosis activity. The pinocytosis activity of the mouse peritoneal macrophages was measured by the neutral red uptake method, which is quantitative spectrophotometric determination of neutral red in macrophages. As shown in Fig. 4, while the treatment with CTX alone (MC group) significantly reduced the phagocytosis activity of the macrophages, the treatment with KLDS1.0318 or levamisole hydrochloride significantly restored it (*p* < 0.05). The effect of KLDS1.0318 [5 × 10^7^ and 5 × 10^8^ CFU/mL (0.2 mL/d)] was comparable to that of levamisole hydrochloride, and there was no significant difference between them (*p* > 0.05). However, the high dose of KLDS1.0318 exhibited a stronger effect on the phagocytic activity of macrophages than that of the PC (levamisole hydrochloride–treated mice) group (*p* < 0.05).

### Effect of L. plantarum KLDS1.0318 on cytokine production

To evaluate the effect of *L. plantarum* KLDS1.0318 on cytokine production, the levels of cytokines IL-2, IL-6, and IFN-γ in mouse serum were examined. As demonstrated in Table 2, compared with the other five groups, CTX injection caused significant reduction in the concentrations of cytokines (IL-2, IL-6, and IFN-γ) in the MC group (*p* < 0.05). Simultaneously, our results showed that the levels of all the cytokines in the KLDS1.0318 treatment groups (L, M, H) were significantly decreased in a dose-dependent manner when compared with those of the MC group (*p* < 0.05). Furthermore, the levels of IL-2, and IFN-γ in the KLDS1.0318 treatment indicated more significant increase (*p* < 0.05) than those of the PC group at the medium and high dose levels (M, H), while the IL-6 levels in the KLDS1.0318 treatment at the high dose group were markedly enhanced when compared with those of the PC group (*p* < 0.05).

**Table 2:** Effect of *L. plantarum* KLDS1.0318 on levels of cytokines in serum in mice

Group	IL-2 (pg/mL)	IL-6 (pg/mL)	IFN-γ (pg/mL)
NC	58.2 ± 2.7^b^	33.2 ± 1.1^c^	82.3 ± 3.2^b^
MC	34.4 ± 2.5^a^	15.4 ± 1.2^a^	60.1 ± 2.8^a^
PC	64.5 ± 1.3^c^	31.4 ± 1.7^c^	87.5 ± 3.7^bc^
KLDS1.0318-L	59.3 ± 1.7^b^	20.7 ± 1.6^b^	92.4 ± 2.9^c^
KLDS1.0318-M	71.7 ± 2.2^d^	32.8 ± 2.1^c^	102.4 ± 4.1^d^
KLDS1.0318-H	79.6 ± 2.3^e^	37.4 ± 1.9^d^	123.5 ± 2.5^e^

NC, non-immunosuppression + sterilized PBS; MC, immunosuppression + sterilized PBS; PC, IM + levamisole hydrochloride (40 mg/kg); KLDS1.0318-L, IM + 5 × 10^7^ CFU/mL *L. plantarum* KLDS1.0318; KLDS1.0318-M, IM + 5 × 10^8^ CFU/mL *L. plantarum* KLDS1.0318; KLDS1.0318-H, IM + 5 × 10^9^ CFU/mL *L. plantarum* KLDS1.0318. Data are expressed as the mean ± SD (*n* = 15). Significant differences (*p* < 0.05) between the groups are indicated with different letters above the data.

## Discussion

As an alkylating agent, CTX has been most extensively used in chemotherapy. Along with a significant clinical effect, it can significantly damage the structure of DNA, impair immune cells, and strongly interfere with the proliferation and differentiation of T and B cells, reducing the number of normal T and B cells. Meanwhile, it reduces levels of inflammatory cytokines (32, 33), thus suppressing the cellular and humoral immune responses of the organism (9). Accordingly, mice treated with CTX were used as an animal model of an immunosuppressed state to validate the immunoenhancement of *L. plantarum* KLDS1.0318 in this experiment.

In the present study, treatment with CTX (80 mg/kg, i.p.) in mice can notably reduce the body weight and immune organ index, inhibit the proliferation of spleen cells, and lower the phagocytosis activity of the macrophages. Moreover, the levels of cytokines IL-2, IL-6, and IFN-γ were decreased by CTX. These experimental data are in accord with previous reports (26, 32, 34). The results described above expressly indicated that the immune functions of BALB/c mice were significantly repressed by CTX and suggested that the immunosuppressive model of mice was successfully established.

Lactic acid bacteria, such as *Lactobacillus acidophilus and Bifidobacterium bifidum*, have been reported to influence one or more components of humoral, cellular, or activate nonspecific immunity (35, 36). For example, it was shown that *Lactobacillus casei* and *L. acidophilus* were capable of enhancing the number of IgA-producing plasma cells *in vivo* in a dose-dependent manner. Probiotics also promoted splenocyte proliferation in response to mitogens for T and B cells in mice and increased the cytokine production of TNF-α, IL-1β, IL-6, and IFN-γ in immune cells (37). Hence, we tested the ability of lactobacillus to reinstate CTX-induced immunosuppression in mice.

The impact of *L. plantarum* KLDS1.0318 on the thymus and spleen indices was determined first, since the thymus and spleen are such important immune organs in the body and the places of growth and proliferation of immunological cells. The thymus is the immune organ in which T lymphocytes develop, proliferate, differentiate, and mature, whereas the spleen mainly contains T and B cells. Consequently, the immune organ index is usually used to reflect the growth of immune organs and evaluate the immunoregulatory effect of probiotics (38, 39). Li et al. (40) reported that some lactobacilli significantly improved the immune organ index. In the present study, our results showed that the thymus and spleen indexes in four treatment groups were obviously greater than those in the MC group at the end of the experiment. The results indicated that *L. plantarum* KLDS1.0318 could resist the influence of immunosuppression on the development of immune organs.

Lymphocyte proliferation in response to corresponding mitogens is commonly determined when evaluating the efficacy of immunomodulatory agents; thus, lymphoproliferation assays have been used to analyze the effects of probiotics on immune function (41). Ren et al. (30) tested the immunomodulatory effects of *Lactobacillus salivarius* CICC 23174 and *L. plantarum* CGMCC 1.557 and found that the splenocyte proliferation index was prominently increased by the two strains in a dose-dependent manner. To test the effect of *L. plantarum* KLDS1.0318 on the cellular immune response, we isolated the splenocytes of mice and examined their proliferation and the activity of NK cells. Our results showed that *L. plantarum* KLDS1.0318 markedly enhanced splenocyte proliferation and the activity of NK cells, suggesting that the strain could improve humoral immunity and cell-mediated immunity and correspondingly could have potential immune activity. We also examined phagocytosis of macrophages in response to *L. plantarum* KLDS1.0318, given that phagocytosis by macrophages is the primary line of defense of the immune system to defend against microbial attack. The results showed a strong increase in phagocytic activity, indicating that *L. plantarum* KLDS1.0318 played a crucial role in the initiation and modulation of nonspecific immune responses by some cytokines and reactive intermediates secreted by macrophages, which is consistent with a previous report (42).

Given that cytokines play a significant role in the development of immune response, the effect of the lactobacillus strain on the production of IL-2, IL-6, and IFN-γ was evaluated. IL-2 is a cytokine essential for the survival and proliferation of T cells. Accordingly, IL-2 secretion increased by KLDS1.0318 may stimulate T-cell proliferation and IFN-γ production, which in turn improves the immune response against cancer and pathogen-infected cells. IL-2 also induces NK cell activation, which restrains the growth and metastases of tumors (43). IL-6 secreted by Th2 cells can regulate humoral immunity (44) as one of the most essential immune and inflammatory mediators that modulate diverse cell functions, such as proliferation and differentiation of B and T cells (45). IFN-γ released by Th1 cells can effectively mediate cellular immunity (46) as one of the dominant immunoregulatory molecules that enhance potent immune responses against pathogenic bacteria and exogenous infectious agents (47). Investigation by Jang et al. (48) showed that *L. casei* HY7213 can induce the production of antitumorigenic cytokines (e.g. IL-2 and IFN-γ). In this study, the concentrations of the three cytokines in each of the KLDS1.0318 dose groups were much higher than those in the MC group. This indicated that *L. plantarum* KLDS1.0318 could highly induce the secretion of some cytokines and help maintain a balance between Th1 and Th2 type cytokines, which play an important role in host immunity.

In summary, the present study has demonstrated that *L. plantarum* KLDS1.0318 improved immunity by promoting immune organ development; enhancing lymphocyte proliferation; increasing the activity of NK cells; improving the activity of macrophage phagocytosis; and upregulating the levels of IL-2, IL-6, and IFN-γ. Consequently, these results suggest that *L. plantarum* KLDS1.0318 is an effective immunomodulating agent and may be effectively used to improve the immune function in humans.
